# A dynamic epibiont community associated with the bone-eating polychaete genus *Osedax*

**DOI:** 10.1128/mbio.03140-22

**Published:** 2023-06-29

**Authors:** Shana K. Goffredi, Balig Panossian, Camille Brzechffa, Naomi Field, Chad King, Giacomo Moggioli, Greg W. Rouse, José M. Martín-Durán, Lee M. Henry

**Affiliations:** 1 Department of Biology, Occidental College, Los Angeles, California, USA; 2 School of Biological and Behavioural Sciences, Queen Mary University of London, London, United Kingdom; 3 Monterey Bay National Marine Sanctuary, Monterey, California, USA; 4 Scripps Oceanography, University of California, La Jolla, California, USA; Max Planck Institute for Marine Microbiology, Bremen, Germany

**Keywords:** *Osedax*, epsilonproteobacteria, epibiont, whalefall, symbiosis, metagenomics, Campylobacterales

## Abstract

*Osedax,* the deep-sea annelid found at sunken whalefalls, is known to host Oceanospirillales bacterial endosymbionts intracellularly in specialized roots, which help it feed exclusively on vertebrate bones. Past studies, however, have also made mention of external bacteria on their trunks. During a 14-yr study, we reveal a dynamic, yet persistent, shift of Campylobacterales integrated into the epidermis of *Osedax*, which change over time as the whale carcass degrades on the sea floor. The Campylobacterales associated with seven species of *Osedax*, which comprise 67% of the bacterial community on the trunk, appear initially dominated by the genus *Arcobacter* (at early time points <24 mo), the *Sulfurospirillum* at intermediate stages (~50 mo), and the *Sulfurimonas* at later stages (>140 mo) of whale carcass decomposition. Metagenome analysis of the epibiont metabolic capabilities suggests potential for a transition from heterotrophy to autotrophy and differences in their capacity to metabolize oxygen, carbon, nitrogen, and sulfur. Compared to free-living relatives, the *Osedax* epibiont genomes were enriched in transposable elements, implicating genetic exchange on the host surface, and contained numerous secretions systems with eukaryotic-like protein (ELP) domains, suggesting a long evolutionary history with these enigmatic, yet widely distributed deep-sea worms.

**IMPORTANCE:**

Symbiotic associations are widespread in nature and we can expect to find them in every type of ecological niche. In the last twenty years, the myriad of functions, interactions and species comprising microbe-host associations has fueled a surge of interest and appreciation for symbiosis. During this 14-year study, we reveal a dynamic population of bacterial epibionts, integrated into the epidermis of 7 species of a deep-sea worm group that feeds exclusively on the remains of marine mammals. The bacterial genomes provide clues of a long evolutionary history with these enigmatic worms. On the host surface, they exchange genes and appear to undergo ecological succession, as the whale carcass habitat degrades over time, similar to what is observed for some free-living communities. These, and other annelid worms are important keystone species for diverse deep-sea environments, yet the role of attached external bacteria in supporting host health has received relatively little attention.

## INTRODUCTION

Whalefalls create a unique environment for deep-sea organisms as the decaying carcass serves as a bountiful, albeit ephemeral, source of nutrition on the seafloor. *Osedax* “bone-eating worms” specialize in these habitats by infiltrating and degrading the whalebones using a unique root-like tissue that contains obligate intracellular symbionts within the Oceanospirillales (1 - 3). This symbiosis has a profound influence on accelerating the degradation of marine mammal skeletons and therefore nutrient remineralization and ecosystem longevity in the deep sea (4). While many studies have examined the diversity, genomics, and physiology of the primary intracellular symbiont of the nearly 30 known species of *Osedax* (e.g., 5 - 9), much less is known about other bacteria, including the Campylobacterales, which have been regularly recovered from the external surface of these important residents of deep-sea whalefall ecosystems (2, 10 - 12).

Campylobacterota, formerly known as Epsilon-proteobacteria, are known to oxidize sulfide and other intermediate sulfur compounds and have an affinity for habitats rich in both organics and sulfides, such as hydrothermal vents, methane seeps, and whalefalls (13 - 15). They are now recognized as important players in deep-sea biogeochemical cycles (16 - 18). At whalefalls, in particular, the Campylobacterales can represent up to ~30% of bacterial ribotypes recovered from bone surfaces or sediments, compared with <2% community membership for sediments beyond the influence of the whale carcass (19, 20). It is, however, currently unclear whether the Campylobacterales found on *Osedax* are non-specific transient associations or persistent epibionts of the worm itself.

A remarkable diversity of bacteria forms non-transient associations with eukaryotes, both internally and externally, and can contribute to the health, physiology, behavior, and ecology of their hosts. Bacteria that interact with surface epithelia can play important ecological roles for animal hosts by reducing exposure to harmful compounds and modulating interactions with predators or pathogens, to name a few (21, 22). The physical and chemical properties of a host surface, prevailing conditions of the surrounding seawater (in the case of marine epibionts), as well as interactions among the microbial residents, can all shape this community. Due to their different ecologies, surface-associated bacteria are often metabolically distinct from their free-living populations, demonstrating higher enzymatic activity, growth and reproduction, and increased lateral gene transfer compared to free-living cells (21, 23). Here, we present a 14-yr study of the bacterial communities associated with the external surfaces of seven species of *Osedax* worms. Using molecular, metagenomic, and microscopy analyses, we reveal a dynamic community of Campylobacterales epibionts associated with *Osedax* that are unique from close relatives and appear metabolically suited to the different stages of whale decomposition.

## RESULTS

**Fig 1:**
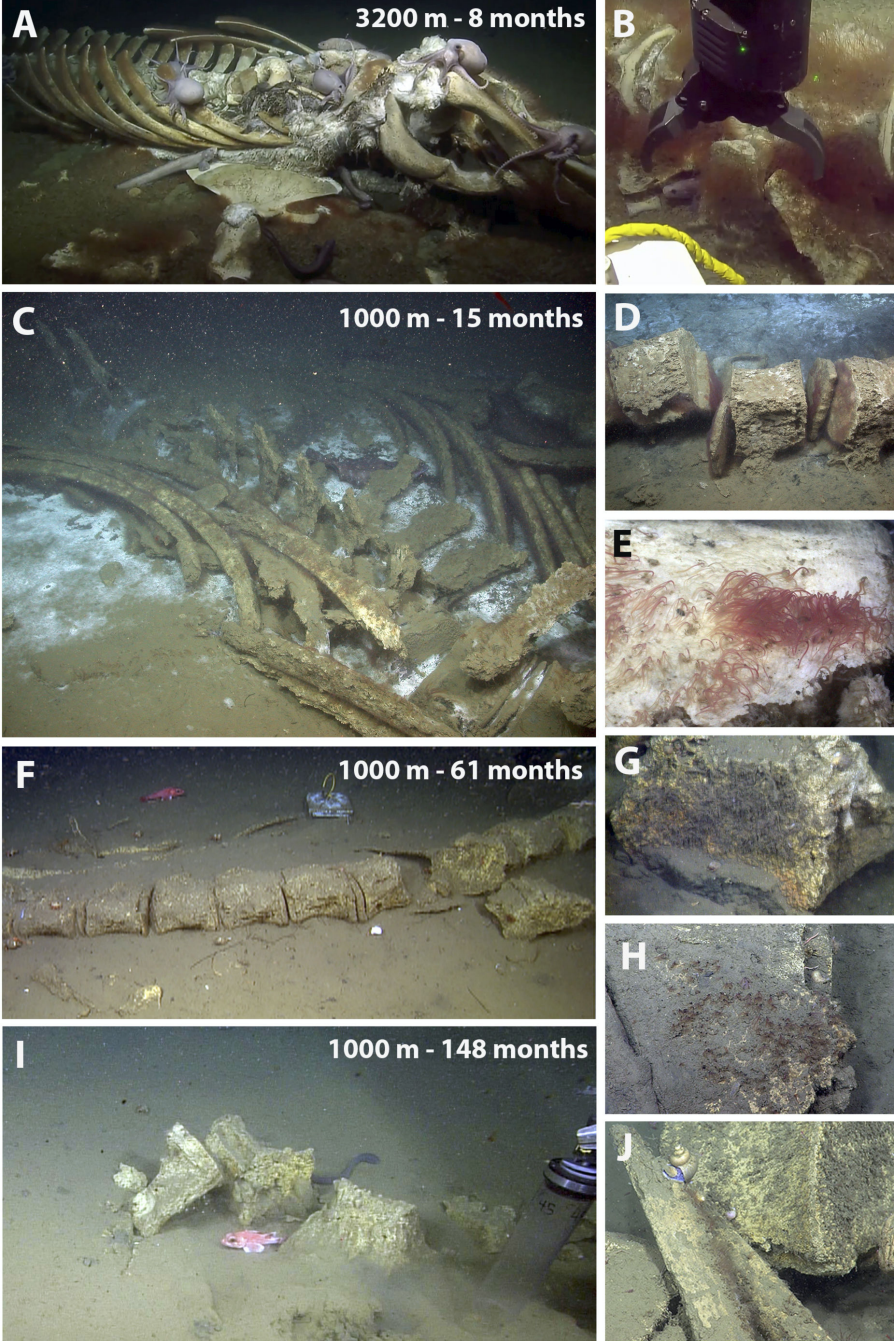
Still images of whalefalls off of northern California, USA, showing decomposition over time and condition of the carcasses at the time of sampling. A 3,239 m whalefall at (**A and B**) ~8 mo (dive H1796, 16 October 2019, initial observation). A 1,018 m whalefall at (**C and E**) 13–15 mo since deposition on the seafloor (dives T916 and T931, 7 November 2005 and 4 January 2006, respectively); (**F and G**) 61 mo (dive DR095, 18 November 2009); (**H and I**) 148 mo (dive DR928, 23 February 2017); and (**J**) 172 mo (dive DR1112, 7 January 2019). H = ROV Hercules, T = ROV Tiburon, DR = ROV Doc Ricketts.

**Fig 2:**
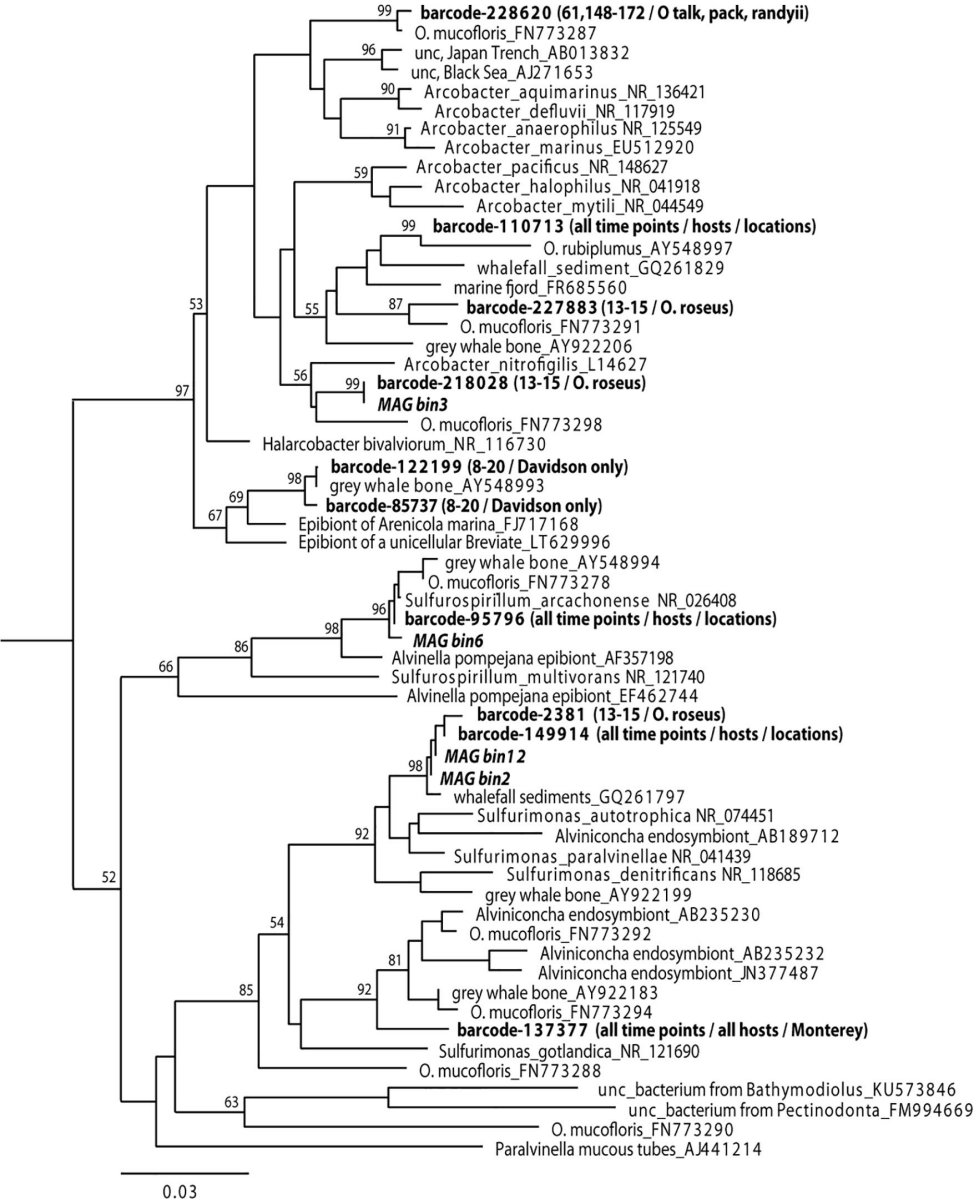
Phylogenetic relationships among the *Osedax* Campylobacterales epibionts, based on the 16S rRNA gene. Taxa in bold denote those generated in this study, along with months of collection. *Helicobacter ganmani* (NR_024836) was used as the outgroup (not shown). Numbers at nodes indicate bootstrap support (1,000 replicates, neighbor-joining, Tamura-Nei model), aligned using Geneious Prime 2021.2.2. Additional sequences from cultured representatives were obtained from GenBank, as were sequences from references 11, 20, and 25.

To characterize the *Osedax*-associated bacterial diversity, we performed 16S rRNA gene amplicon sequencing of 37 specimens collected from two Pacific Ocean sites (Table 1; Fig. 1). Based on this analysis, the Campylobacterales was identified as the dominant bacterial group associated specifically with the *Osedax* trunk (67 ± 19%; Fig. S1). *Arcobacter*, *Sulfurospirillum,* and *Sulfurimonas* were the primary *Osedax*-associated Campylobacterales genera recovered, and specific ribotypes were distinct from those known to associate with other animals from reducing habitats (only 82% 16S rRNA gene similarity; Fig. 2). The only other common bacterial 16S rRNA gene amplicon was from an uncultured member of the Kordiimonadales (Alphaproteobacteria; comprising 29 ± 17% of the microbial community; Fig. S1), closely related to those recovered previously from the external surface of *Osedax* (11) and sunken wood (24).

**TABLE 1:** Sample locations, along with dive information, time frame, and *Osedax* species identities for specimens used in this study

Whale	Dive no.^*d*^	Date	Time frame (months)	*Osedax* species present
Davidson^*a*^^,^^*f*^ (3,239 m)	H1796	Oct 2019	8	n.sp
	H1825	Oct 2020	20	*lonnyi*
Monterey^*b*^ (1,018 m)	T916	Nov 2005	13	*roseus*
	T919	Nov 2005	13	*roseus*
	T931	Jan 2006	15	*roseus*
	T1049	Oct 2006	24	*packdorum*
	DR009	Mar 2009	52	*talkovici*
	DR095	Nov 2009	61	*talkovici*
	DR928	Feb 2017	148	*packdorum*
	DR966	July 2017	154	*packdorum*
	DR1029	May 2018	164	*packdorum/talkovici*
	DR1105	Dec 2018	171	*packdorum/talkovici*
	DR1112	Jan 2019	172	*packdorum/talkovici/randyi*
Monterey^*c*^^,^^*f*^ (2,891 m)	T769	Nov 2004	34^*e*^	*frankpressi*
	T991	May 2006	51	*frankpressi*

^*a*^ Natural whalefall discovered October 2019 (35.582ºN/122.629ºW).

^*b*^ Artificial whalefall implanted October 2004 (36.772ºN/122.083ºW).

^*c*^ Natural whalefall discovered February 2002 (36.613ºN/122.434ºW).

^*d*^ H = ROV Hercules, T = ROV Tiburon, DR = ROV Doc Ricketts.

^*e*^ Used for metagenomics only.

^*f*^ Both natural whalefalls were estimated to have been on the seafloor at least 8 mo, based on tissue condition, which was remarkably similar (compare Fig. 1 to Fig. 1 in reference 1; time frame = initial visit + 8 mo).

**Fig 3:**
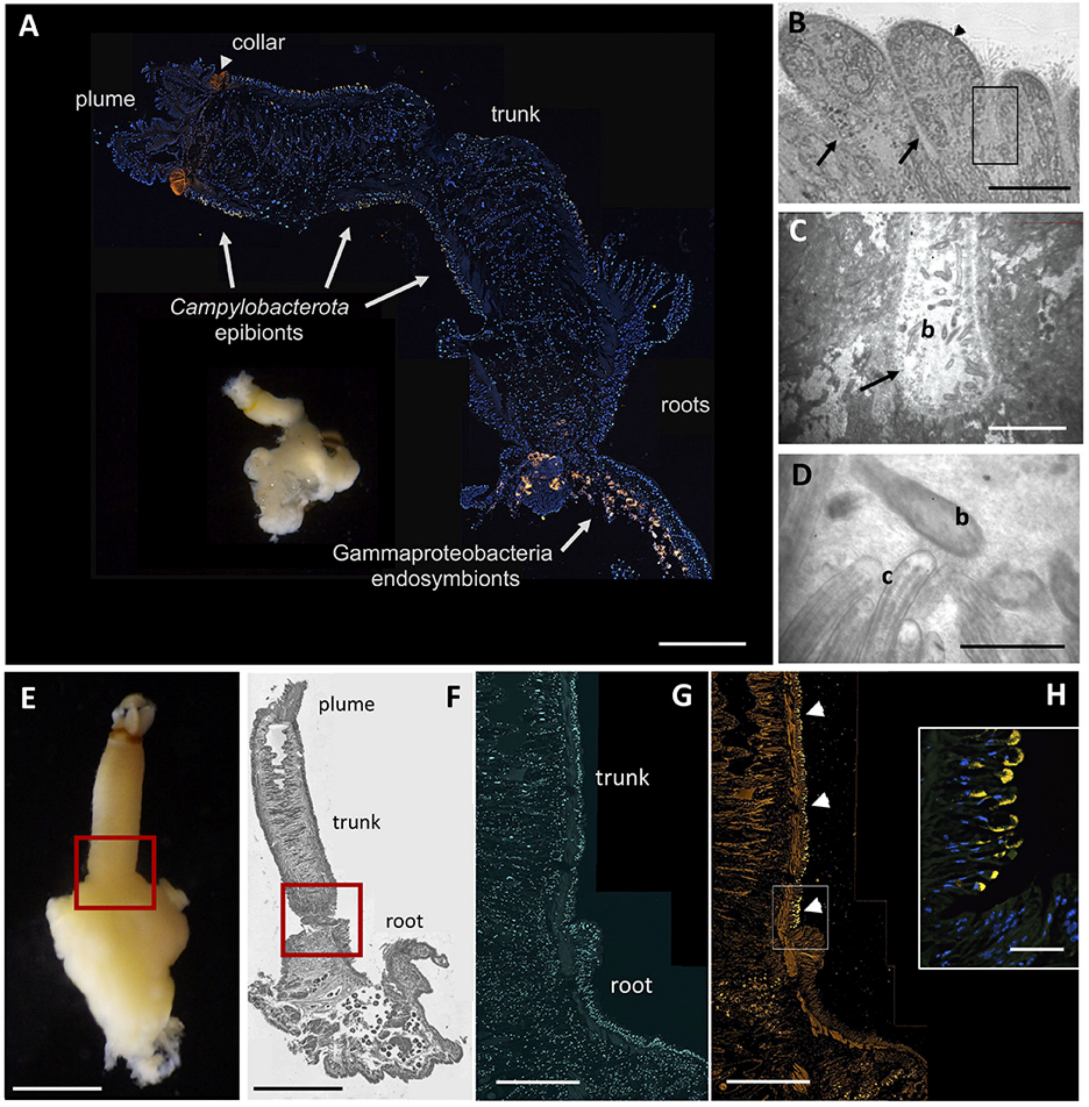
Microscopy of *Osedax packardorum*. (**A**) Whole image of a specimen from dive DR966, side-by-side with a longitudinal cross section, from plume to roots, hybridized with a fluorescent probe targeting all bacteria (Eub338_Cy3, shown in orange), and counterstained with DAPI, showing host cell nuclei in blue. (**B - D**) Transmission electron (TEM) microscopy of *Osedax* trunk tissue (dive DR1112), revealing bacteria-like cells (b) in epidermal grooves (arrows), in contact with host cilia (c, arrowhead). Square in B highlights regions in C and D. (**E**) Image of whole specimen from dive DR1105. (**F**) Light microscopy of 5 µm Wright-stained section embedded in Steedman’s resin. (**G and H**) Paired images showing the signal from DAPI (**G**) and a fluorescent 16S rRNA probe targeting the Campylobacterales, EPS549_Cy3 (**H**), revealing the abrupt delineation in epibiont presence (at arrowheads) between the trunk and roots, with slight autofluorescence. (inset) FISH microscopy using probes EPS549_Cy3 and Eub338_Alexa488. Complete overlap between the probes is shown in yellow, in addition to DAPI-stained host cell nuclei in blue. Scale bars: A, 200 µm (not including inset). B, 100 µm. C, 25 µm. D, 1 µm. E, 500 µm. F, 300 µm. G-H, 500 µm. Inset, 50 µm.

**Fig 4:**
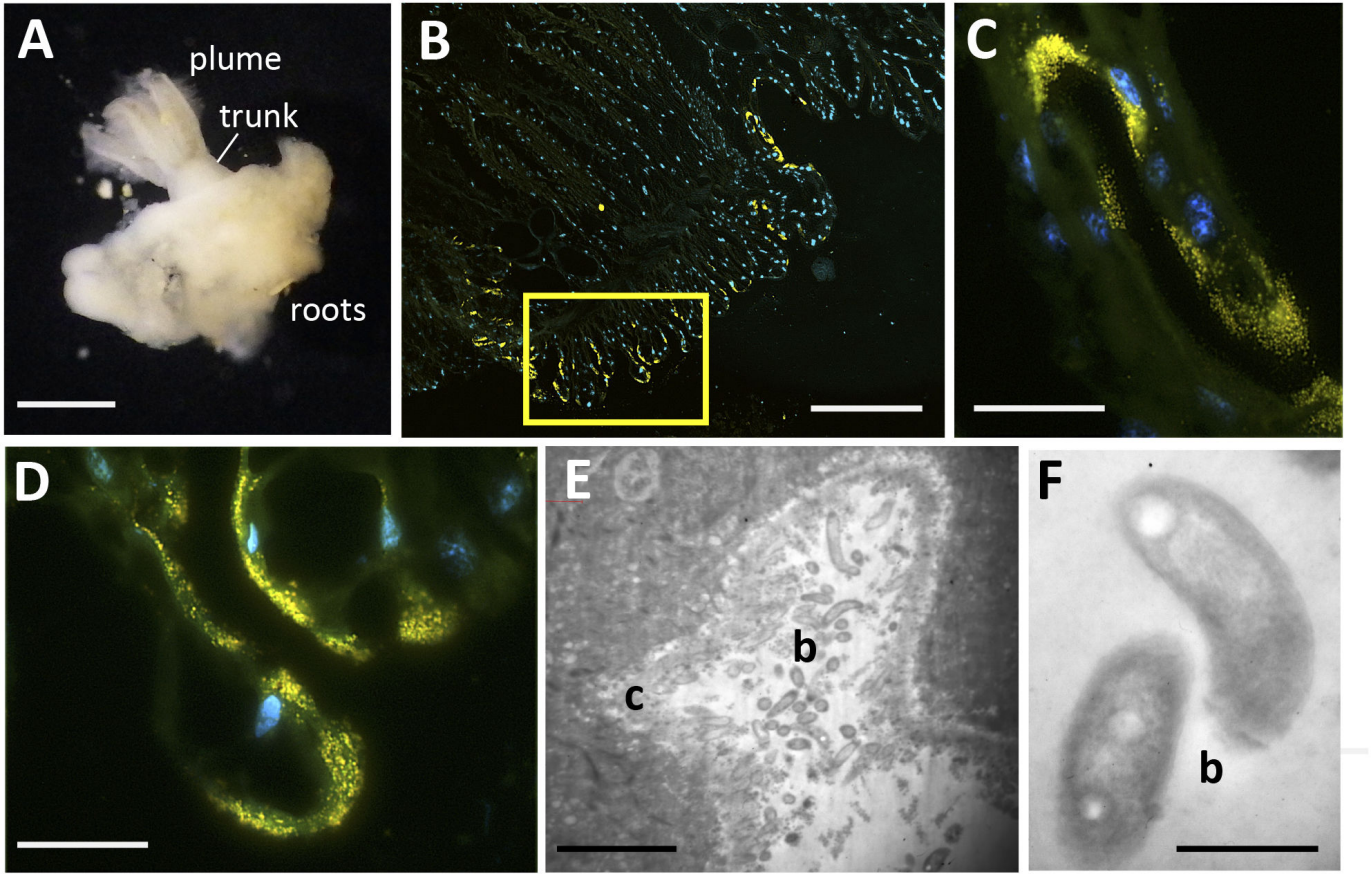
Microscopy of *Osedax talkovici*. (**A**) Whole image of a specimen from dive DR1112. (**B - D**) Fluorescent *in situ* hybridization (FISH) microscopy using probes Eub338_Alexa488 and EPS549_Cy3. Complete overlap between the probes is shown in yellow, in addition to DAPI-stained host cell nuclei in blue. (**E and F**) Transmission electron (TEM) microscopy of *O. talkovici* trunk tissue, revealing bacteria-like cells (b) in epidermal grooves, in contact with host cilia (c). Scale bars: A, 500 µm. B, 100 µm. C-D, 10 µm. E, 20 µm. F, 0.5 µm.

Fluorescence microscopy showed a close association of the Campylobacterales with the trunk epithelial surface of *Osedax*. The Campylobacterales were the only obvious bacteria present along the epidermis, based on overlap between universal and specific bacterial probes (Fig. 3 and 4). They occurred along the full length of the trunk and appeared very closely attached to the apical end of exposed epidermal labia, although some also appeared in epidermal cavities, as was seen via TEM (Fig. 4C and D; Fig. S2). Slight autofluorescence of the matrix in which the bacteria were embedded made the determination of their specific position inconclusive via fluorescence *in situ* hybridization (FISH) microscopy. For the mucous tube, which is secreted by numerous glands on the trunk and is used by the worm to glide up and down, only non-Campylobacterales bacteria were identified via microscopy (Fig. S3). No bacteria were observed on plume or root surfaces (Fig. 3A through H), despite significant surface area of both tissues.

**Fig 5:**
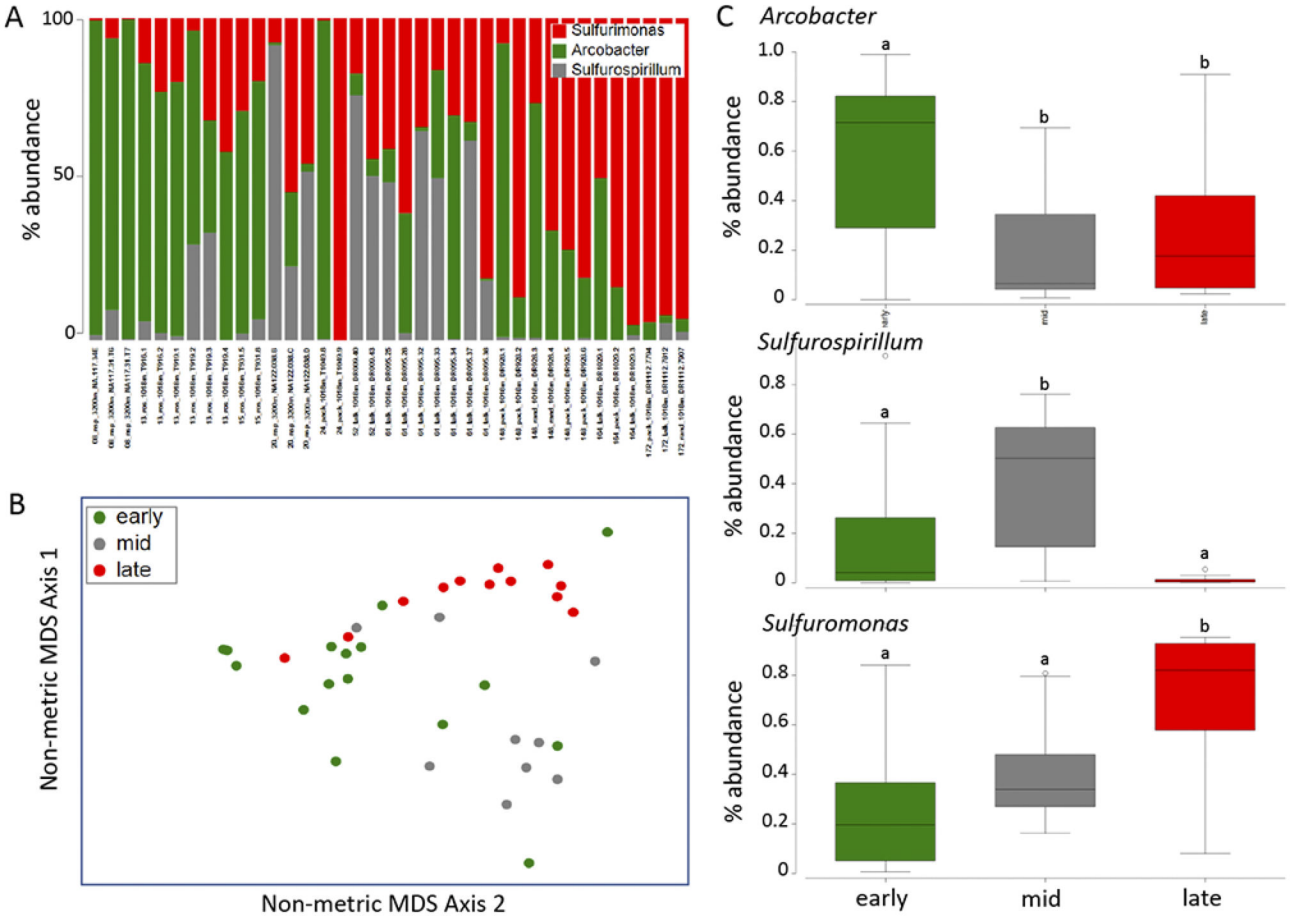
Dominant Campylobacterales 16S rRNA gene amplicon sequences recovered from barcoding of six *Osedax* species at two whalefalls off of northern California, USA. (**A**) Relative abundance of the 16S rRNA gene for the genera *Arcobacter, Sufurospirillum,* and *Sulfurimonas*. (**B**) Non-metric multidimensional scaling (NMDS) ordination of Campylobacterales communities associated with *Osedax* (square root transformation; Bray-Curtis similarity). Each point represents all Campylobacterales 16S rRNA gene sequences recovered from a single specimen. Ordination comparing three different time points from the 1,018 m whalefall; early (13–24 mo), mid (52–61 mo), and late (148–172 mo). ANOSIM *P* < 0.01 for all three comparisons, suggesting a significant difference between timeframes, but with some overlap (R = 0.35–0.53). (**C**) Box plots of the three dominant *Osedax*-associated genera, as relative percent abundance, at two different whalefalls and three different time points: early, mid, and late. (*n* = 16, 9, and 12, respectively). Levels of significance based on ANOVA *P* < 0.05. Data points outside of the 25–75% range are identified by open symbols.

To explore whether *Osedax*’s epibiont community changes as the whale carcass degrades, we collected the annelids from two whalefalls between 8 and 172 mo (>14 yr) after carcass deposition; one intentionally deposited on the seafloor at 1,018 m depth in the Monterey Canyon (4) and a second discovered in an early stage of decomposition at 3,239 m depth on the Davidson seamount, both off the coast of California (Table 1; Fig. 1). Regardless of *Osedax* species, *Arcobacteraceae* were the dominant microbial group associated with the worm trunks at early time points (<24 mo, *n* = 16), constituting 59% of the average recovered Campylobacterales ribotypes, a significantly higher representation than at later time points (18–28%; ANOVA *P* < 0.02; Fig. 5). *Osedax* collected at early time points had relatively few aggregations of bacteria along the trunk that were visible by microscopy, while at later time points bacteria appeared to cover much more of the epithelial surface (Fig. 3 and 4, compared to Fig. S4). This trend was supported by quantitative PCR (QPCR) data that revealed 1.2 × 10^4^ bacteria per ng DNA, on average, for *Osedax* from the early-mid time points (*n* = 4; dives T916, T919, T931, and DR095) and 4.5 × 10^5^ bacteria per ng DNA for *Osedax* from later time points (*n* = 2; dive DR1029). For reference, the root tissue of a single individual from dive DR1029 hosted 2.1 × 10^6^ bacteria per ng DNA, presumably entirely comprised of the primary symbiont. A specific *Arcobacter* ribotype occurred at the Davidson site and was related to those associated with other eukaryote hosts (Fig. 2). *Sulfurospirillum* appeared to peak in abundance during the mid-stages of whalefall degradation (~50–60 mo, *n* = 9), representing 41% of the recovered Campylobacterales ribotypes, compared to 12–16% at early and late stages (ANOVA *P* < 0.03; Fig. 5). While at later time points (>140 mo, *n* = 12) the dominant genus transitioned significantly to *Sulfurimonas,* averaging 71% of Campylobacterales ribotypes compared to early-mid time periods (25–40%; ANOVA *P* < 0.01; Fig. 5). All observed *Sulfurospirillum* and *Sulfurimonas* ribotypes were shared among *Osedax* species, regardless of Davidson or Monterey Canyon sites, suggesting that neither host species nor seafloor location, even at vastly different depths, plays a major role in assembling the specific *Osedax* Campylobacterales community (only 20–30% of the variation was influenced by either factor, according to one-way ANOSIM; Fig. S5). We note that after metagenomic sequencing, described below, each of the three Campylobacterales genera had different rRNA copy numbers (ranging from 2 to 12; Table 2). A normalization of the barcode data, taking into consideration these differences, did not change the general trends in abundance shifts over time (Fig. S6).

**TABLE 2:** *Osedax*-associated epibionts genome sizes, coding sequences, depth of coverage, completeness (based on BUSCO and CheckM values), contamination, and Average Nucleotide Identity (ANI) values showing degree of similarity to closest relatives with available genomes in GenBank

Taxon	Coverage	No. of contigs	Genome size (bp)	N50	rRNA copy no.	BUSCO completeness	CheckM completeness	CheckM contamination
*Arcobacter* epibiont	34×	1	2,901,687	2,901,687	6	93.1	93.5	6.79
*Sulfurospirillum* epibiont	13×	33	2,726,073	225,838	2	92.7	95.1	4.27
*Sulfurimonas* epibiont HC	268×	1	2,827,517	2,827,517	12	98.6	100	0.61
*Sulfurimonas* epibiont LC	16×	1	2,607,188	2,607,188	12	95.1	98.7	1.02

To better understand the physiological potential, and therefore ecological influences, of the *Osedax* epibionts, we performed metagenomic sequencing of a single specimen of *Osedax frankpressi* collected from a 3rd whalefall at 2,891 m depth in Monterey Canyon. We identified four near-complete genomes of the dominant *Osedax* epibionts (completeness scores of 93–100%; 0.6–6.8% contamination; Table 2), with nearly identical 16S rRNA gene sequences to those recovered via barcoding (99.6–100%; Fig. 5). These genomes belonged to *Arcobacter* (*sensu lato*, closely related to *Arcobacter nitrofigilis*, the type species of the genus; 26), *Sulfurospirillum* and *Sulfurimonas*, the three dominant Campylobacterales epibionts, the latter represented by two distinct genomes identifiable by a large difference in sequencing coverage depth (16 vs 268×; Table 2). The “high-coverage” *Sulfurimonas* (268×), referred to further in the following metagenomic sections unless otherwise noted, was in far greater abundance than even the well-known primary Oceanospirillales endosymbiont (at 30× coverage). A single Kordiimonadales (Alphaproteobacteria) genome was also recovered from the external surface of *Osedax*; however, our microscopy analysis did not indicate integration into the *Osedax* epithelium, so we do not focus on it further (genome available at # PRJNA813420).

**Fig 6:**
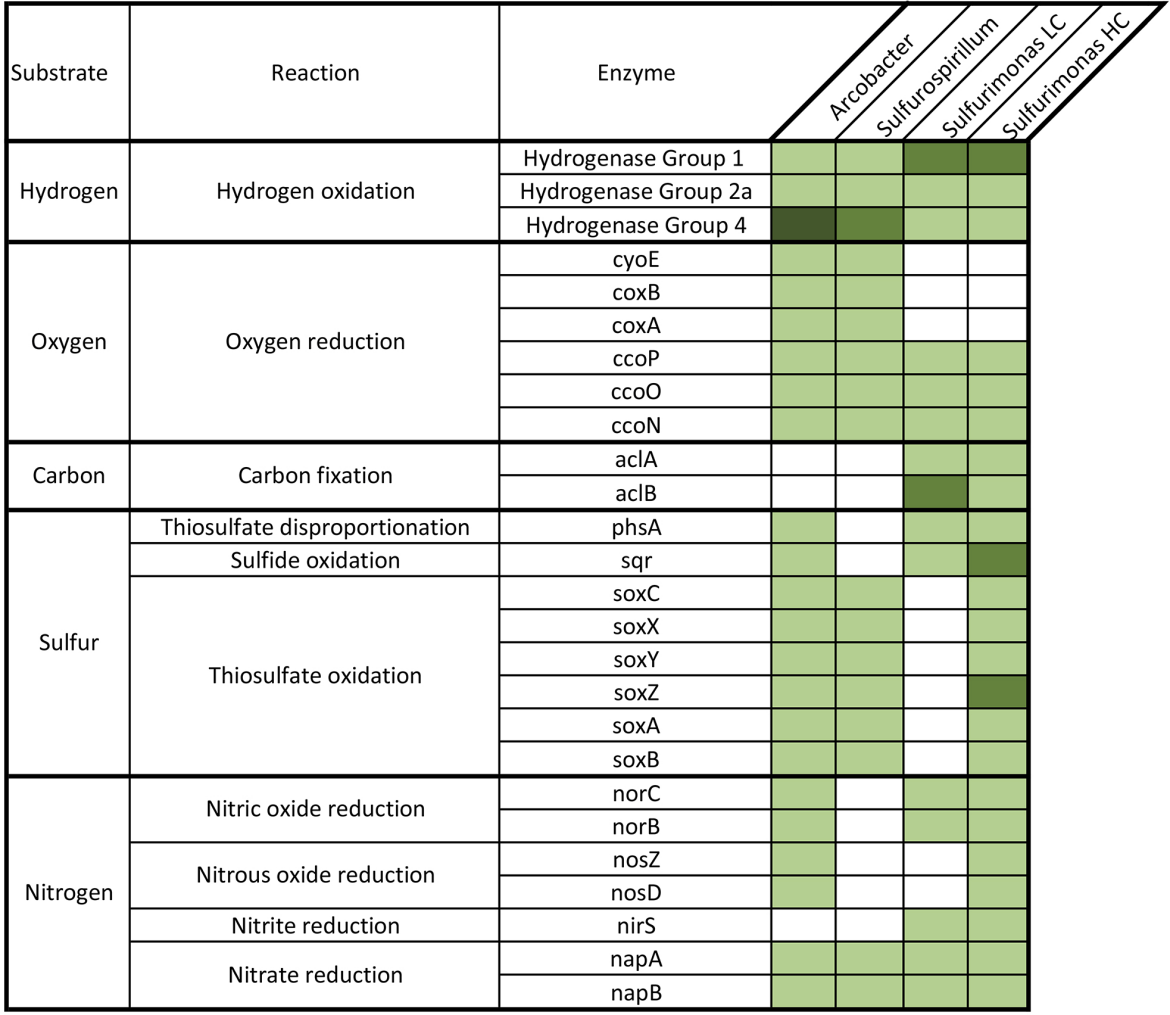
Comparison of genes involved in hydrogen, oxygen, carbon, sulfur, and nitrogen (H, O, C, S, N) metabolism present in the Campylobacterales epibionts associated with *Osedax,* using a Hidden Markov Motif (HMM) gene identification analysis through LithoGenie. The number of matches to the metabolic HMMs were quantified for each sample and visualized as a heatmap, including the copy number of each gene. Compared to one another, the epibionts showed a shift in their degrees of investment to carbon and sulfur metabolism later in the degradation process, when *Sulfurimonus* dominates, reflected by their total number of genes involved in each biochemical process.

An analysis of genomic motifs revealed differences in the metabolic capabilities among the *Osedax* epibionts. In general, there appeared to be a temporal shift in metabolic strategy from heterotrophy to autotrophy, from *Arcobacter* (s.l.) and *Sulfurospirillum* at early-mid time points to *Sulfurimonas* at later time points (Fig. 6) All of the epibionts can metabolize hydrogen using a shared suite of hydrogenase enzymes (Groups 1, 2a and 4), although gene copy numbers vary. The epibionts differ, however, in their capacity to metabolize oxygen, carbon, nitrogen, and sulfur. For oxygen metabolism, *Arcobacter* and *Sulfurospirillum* possess cyoE and the cytochrome oxidase genes coxA/B - involved in processing heme and electron transport during aerobic respiration –which are absent in Sulfurimonas (Fig. 6). All *Osedax*-associated epibionts can reduce nitrate (via napA/B), however, only the *Sulfurimonas* epibionts possessed the nirS gene for nitrite reduction. In addition, the *Arcobacter* and *Sulfurimonas* possessed genes involved in the reduction of nitric/nitrous oxide that are absent in the *Sulfurospirillum*. By contrast, the *Sulfurimonas*, which dominates the trunk surface at later stages of host decomposition, contained genes involved in carbon fixation through the reverse TCA cycle (aclA and aclB; Fig. 6), which were not found in the other epibionts. While all 3 genera can utilize sulfur compounds, the high-coverage *Sulfurimonas* epibiont has additional genes involved in sulfur metabolism, including two copies each of the sulfide:quinone oxidoreductase (sqr) and the soxZ gene, in addition to the full thiosulfate oxidation (sox) pathway. The sox pathway was notably absent from the low-coverage *Sulfurimonas* strain (Fig. 6).

The average amino acid identity of the *Osedax* epibionts differed considerably from close relatives (*N* = 50; 55–78%), suggesting significant divergence time between the epibionts and free-living relatives with available genomes (Table S1). Despite having similar genome sizes to free-living deep-sea lineages, all three *Osedax*-associated Campylobacterales have decreased coding densities, and significantly more transposable elements that represented ~2–6% of their genomes, compared to <1% for all but a few free-living relatives (Table 3; S1). Given the high quality of the genomes used in this analysis (3 epibionts and 40 free-living relatives had genomes on a single contig), the substantial increase in transposable elements in the epibiont genomes is unlikely to be an artefact of assembly quality. A pan-transposase analysis revealed that the epibionts shared numerous insertion sequence families among them; however, they did not share any with the primary symbiont (Table S2). Insertion sequences were not, however, identical at the base pair level, indicating that while epibionts tend to carry the same families of insertion sequences, they are not sharing them on ecological time scales. Functional genes carried on transposons included those that encode for a Type I restriction modification system (*hsdM* superfamily), a leukotoxin export ATP-binding protein *ltxB*, and a toxin of the *relE/parE* family, which were shared by all *Osedax* Campylobacterales. An additional membrane fusion protein (MFP) of a Type 1 secretion system (T1SS) was also identified on a transposon in the high-coverage *Sulfurimonas* epibiont (Table S3). Lastly, all *Osedax* Campylobacterales shared a Mu-like bacteriophage, which was not present in free-living relatives, or in the primary Oceanospirillales endosymbiont. The phage appeared intact in the high-coverage *Sulfurimonas* and was ~19 kb in size, with 15 open reading frames (ORFs), 13 of which code for proteins (7 are known viral proteins and 6 are hypothetical; Table S4), and two insertion sequences (*attL* and *attR*).

**TABLE 3:** Genome highlights from the Campylobacterales bacteria found on *Osedax*, compared to the genomes of six closest relatives, both cultured and uncultured from the deep sea^*a*^

Bacterial ID	Genome size (bp)	No. of secreted proteins	% of SP with ELP	No. of insertion sequences	% IS (by length)
***Arcobacter* epibiont**	**2,901,687**	**349**	**59.3**	**227**	**4.5**
*Arcobacter anaerophilus* (CP041070_0)	3,016,922	159	13.2	10	0.6
*Arcobacter aquamarinus* (CP042812_0)	2,829,476	175	8.0	8	0.5
*Arcobacter butzleri* (CP000361_0)	2,341,251	146	2.7	3	0.4
*Pseudoarcobacter acticola* (CP042652_0)	3,019,071	256	36.7	105	4.2
GCA_000585115.1	2,287,768	185	6.5	7	0.8
GCA_000585155.1	2,496,885	178	12.9	27	1.3
***Sulfurospirillum* epibiont**	**2,726,105**	**313**	**41.5**	**205**	**6.5**
*Sulfurospirillum deleyianum* (GCA_000024885)	2,306,351	97	32.0	35	1.7
*Sulfurospirillum multivorans* (GCA_000568815)	3,175,729	204	48.0	73	2.4
GCA_000265295.1	2,510,109	90	23.3	13	0.9
GCA_002205395.1	2,876,607	176	41.5	8	0.4
GCA_002309535.1	2,814,086	148	35.1	20	0.7
GCA_008083195.1	3,181,530	202	47.5	67	2.3
***Sulfurimonas*** **epibiont (HC)**	**2,827,517**	**255**	**46.3**	**68**	**2.5**
***Sulfurimonas*** **epibiont (LC)**	**2,607,188**	**196**	**30.6**	**41**	**2.2**
*Sulfurimonas autotrophica* (CP002205)	2,153,198	112	20.5	11	1.1
*Sulfurimonas denitrificans* (CP000153)	2,201,561	112	13.4	8	0.9
*Sulfurimonas sediminis* (CP041235_0)	2,320,257	154	39	76	2.9
GCA_000242915.2	2,952,682	321	62	24	0.7
GCA_000445475.1	2,302,023	106	17	9	0.6
GCA_009192995.1	2,093,483	106	14.2	6	0.8

^*a*^ Boldface indicates metagenomes.

The *Osedax*-associated *Arcobacter* and *Sulfurimonas* also possessed additional genes that encode proteins involved in attachment and secretion system machinery (Table S3). For example, the *Arcobacter* contained five copies of the Type 5a secretion system (T5aSS), compared to 0–2 copies in close relatives. The *Sulfurospirillum* epibiont had a tight adherence (TAD) pilus, distinguishing it from close relatives. Similarly, the high-coverage *Sulfurimonas* epibiont had a Type 5a secretion system absent in close relatives and a complete 14-gene Type 6 secretion system (T6SS) containing 15 copies of the *tssD* gene and 21 copies of the *tssI* gene. The *tssD* and *tssI* genes encode 2 of the 12 core T6SS subunits; the stacked hexameric rings (i.e., Hcp tube) that extend outward from the bacterial cell membrane and the distal cell-puncturing device (a trimer of VgrG), respectively.

Finally, the *Osedax* Campylobacterales genomes contained hundreds of genes encoding predicted secreted proteins, and within these, significantly more (31–59%) had eukaryotic-like protein (ELP) domains compared to free-living relatives (2–30×; Table 3; S1). These ELPs, which can be mobilized by the secretion systems described above, comprised 54 families, based on Pfam identification, plus seven others of unknown function (Table S5). Several of the ELPs were shared among the three main *Osedax* Campylobacterales, including ATP:guanido phosphotransferase (N-terminal domain), an integrase core domain, and a Helix-turn-helix (HTH)-like domain. The early colonizers *Arcobacter* and *Sulfurospirillum* also shared a homeobox-like domain, coding proteins in a large family of transcription factors that contain a highly conserved DNA-binding domain and a second integrase core domain. The high- and low-coverage *Sulfurimonas* genomes, not unexpectedly based on their shared evolutionary history, shared many of their ELPs (~50%; Table S5).

## DISCUSSION

Over the course of 14 years, pervasive Campylobacterales epibionts were observed associated with the external surface of seven *Osedax* species from deep-sea whalefalls off of northern California (1,018–3,239 m depth). The persistence of this specific bacterial order, which had been noticed with *Osedax* previously (2, 10, 11), supports the assertion by Verna et al. (11) that this relationship is more than transitory. Metagenome analysis suggests a long-term association between the Campylobacterales and their *Osedax* hosts based on an abundance of genes encoding secretions systems that are absent in free-living relatives, perhaps to ensure attachment to the host, and an enrichment in secreted proteins with ELP domains. ELPs, which can be rare for some groups (e.g., *Arcobacteraceae*), are considered a bacterial strategy for modulating eukaryotic processes and, in a few symbiotic systems, have been implicated in extracellular secretion, cell binding, colonization, and protein-protein interactions (27 - 29). Some of these domains may even encode signal peptides that interact with secretion systems (30), which were also observed in the *Osedax*-associated Campylobacterales epibionts. ELPs can either be acquired by horizontal gene transfer from a eukaryote, followed by divergent evolution, or through convergent evolution with a non-homologous protein, both of which would take time to evolve. Additionally, they also shared a Mu-like bacteriophage carrying numerous unknown genes. The pronounced abundance of mobile elements in the *Osedax*-associated Campylobacterales, including toxin genes shared between them, suggests a dynamic transfer of genetic material between the microbes, either via cell-to-cell contact or phage transfer.

The relationship between *Osedax* and the Campylobacterales is not fixed, as the trunk epidermis is repeatedly exposed and recolonized throughout the course of whalefall degradation. Temporal succession has been observed for the *Osedax* host species (4) and their primary symbionts (7, 8), as well as free-living microbial communities in the surrounding sediments (9) as the whale carcass degrades, suggesting a direct influence of the local environment on associated microbial populations (7). While *Osedax* host species in this study did not appear to influence the dominant epibiont type, we note that during whale decomposition there is a noticeable shift in *Osedax* species (4) that requires further investigation. Along the trunk epidermis of *Osedax, Arcobacteraceae* was the dominant founding bacterial group, despite differences in water depth or seafloor location. Similar ribotypes were also recovered from *O. roseus* collected at 3 mo from the 1,018 m depth in Monterey Canyon sampled in this study (10) and *O. mucofloris* collected from a Minke whalefall off the coast of Sweden, at 36 mo post-implantation (11, 31), a relatively early time point in whalefall degradation. The *Arcobacteraceae* is a familiar early colonizer in sulfur-rich habitats (32, 33). They have also been identified as pioneer producers of floc during *in situ* and shipboard experiments with bacterial biomass collected from hydrothermal vents at 9°N East Pacific Rise (34). Additionally, a recent study demonstrated that the microbial community composition of *Arcobacter, Sulfurimonas*, and *Sulfurovum* in hydrothermal vent fluid incubations was highly dependent on oxygen levels (35), a parameter that also varies dramatically during whalefall decomposition (36). A general predisposition for oxic environments by the *Osedax*-associated *Arcobacter* is indicated by the possession of the heme O synthase gene *cyoE* and the cytochrome oxidase genes *coxA/B*, involved in electron transport during aerobic respiration. The *Osedax*-associated *Arcobacter* (s.l). is a heterotroph, likely dependent on organic substrates available on the host surface. Similar to the *Arcobacter* recovered from *Lenisia*, a Breviatea protist, the *Osedax*-associated *Arcobacter* possesses numerous hydrogenase genes and those involved in a cellular response to nitric/nitrous oxide, which underpin the mutual benefits in the *Lenisia-Arcobacter* symbiosis through the transfer of hydrogen (37). The relevance of this capability as a symbiont specificity determinant, as observed in other symbioses (38), remains unconfirmed for both the *Osedax*-associated *Arcobacter* and *Sulfurimonas*.

*Sulfurospirillum* was associated with *Osedax* trunk surfaces throughout the duration of this study, but was most prominent during the intermediate time frames from ~50 to 60 mo, suggesting some adaptability during the transitional stages of organic carbon breakdown. This genus is globally found in deep-sea habitats rich in sulfur compounds (39), and the *Osedax*-associated phylotype in the 16S rRNA gene was similar to those found previously in whalefall environments (2, 20). The only other host-associated *Sulfurospirillum* described thus far is a heterotrophic, hydrogen-utilizing epibiont of the vent worm *Alvinella pompejana* (40). Unlike the *A. pompejana* epibiont, the *Osedax*-associated *Sulfurospirillum* is missing the *phsA* and *sqr* genes, so must rely on exogenous thiosulfate (40). This may explain why it never dominated the *Osedax* trunk community by itself but rather co-occurred with either *Arcobacter* or *Sulfurimonas*, both of which can oxidize sulfide to thiosulfate. Additionally, the genome of the *Osedax*-associated *Sulfurospirillum*, unlike close relatives, possesses a gene cluster encoding a TAD pilus, the adhesive structure often used for colonization of surfaces, including eukaryote hosts (41, 42).

The chemoautotrophic *Sulfurimonas* dominated the epibiont community associated with *Osedax* during later stages of whale decomposition (>140 mo). Via metagenomic analysis, two *Sulfurimonas* genomes were recovered, one of which was far more abundant than even the primary intracellular Oceanospirillales symbiont, based on a tenfold higher genome coverage depth. The hydrogen and sulfur-utilizing capabilities of the autotrophic *Osedax*-associated high coverage *Sulfurimonas* appears to be similar to two other Campylobacteria, *S. paralvinellae* and *S. hydrogeniphila*, isolated from a deep-sea tubeworm “nest” and a hydrothermal vent chimney, respectively (25, 43). A community shift from *Arcobacter* and *Sulfurispirillum* (both generally organotrophs) to autotrophic *Sulfurimonas* species is likely influenced by changes in the chemical environment of decomposing organic matter. Kalenitchenko et al. (33) noted a temporal transition from chemoorganotrophic metabolism to chemoautotrophic reliance in reduced deep-sea wood mesocosms (33). A similar shift from *Arcobacter* to *Sulfurimonas* at hydrothermal “snowblower floc” eruptions has been attributed to elevated hydrogen sulfide levels and the subsequent utility of both the *sox* and *sqr* systems by *Sulfurimonas* (44). Indeed, the high-coverage *Osedax*-associated *Sulfurimonas* genome possessed type II and type IV sulfide:quinone oxidoreductase genes, which encode a key enzyme involved in sulfide homeostasis (oxidation and assimilation) and detoxification in bacteria (43), and may help protect its host from harmful by-products during late stages of whale decomposition. Dominance of the high coverage *Sulfurimonas* at later stages of whale carcass decomposition may be due to the numerous reduced sulfur compounds the bacteria can use as energy sources (45), and deployment of an arsenal of secretion systems, which are often used to form biofilms and gain a competitive advantage over neighboring bacteria, as observed in both the *Euprymna* squid and legume symbioses (46, 47).

With the exception of obvious nutritional episymbioses, such as ciliates, nematodes, and yeti crabs (48 - 52), the role of attached external bacteria in supporting host health has received relatively little attention. With regard to *Osedax*, Borchert et al. proposed that the host could benefit nutritionally from enhanced dissolution of inorganic bone components by proton release and subsequent acidification by the sulfur-oxidizing epibionts (53). This appears unlikely based on the distinct lack of epibiotic bacteria on the root surfaces, the only tissue in contact with the bone. Our analysis suggests that detoxification of sulfide by the bacteria may be a possible benefit to the *Osedax* worm host. Hydrogen sulfide is likely to emanate from the whale carcass, especially at later stages of decomposition, and thus sulfide-oxidizing bacteria positioned near the tissue-bone interface could convert this sulfide to less toxic products. Bacteria associated with epithelial surfaces produce metabolic by-products that can be absorbed across the epithelial barrier, thereby influencing the host (54), however, whether the Campylobacterales bacteria of *Osedax* are commensal or beneficial remains undetermined.

### Conclusion

Ecological factors shaping the epibiont communities of marine organisms remain poorly understood. The recurrence of three Campylobacterales genera associated with diverse *Osedax* species collected from multiple deep-sea locations suggests they are specific epibionts that share a long-evolutionary history with their host. All three epibiont types have an affinity for organic-rich and sulfide-rich habitats, however, a notable shift in their composition reveals that they are a dynamic community that changes over time. Factors shaping the epibiome may include the metabolic capabilities of the bacteria themselves, host-controlled changes to the epidermis, and the chemically diverse abiotic conditions (e.g., sulfides, oxygen, and nutrients) that change as the whale carcass degrades over time. Our metagenomic analysis revealed the *Osedax*-associated Campylobacterales to possess genes that allow them to both fuse with the host epithelium and subsequently take advantage of the metabolic opportunities in their changing environment while attached to a host. The presence of extensive secretion systems may also influence their composition, by moderating interactions with *Osedax* and/or competing microbes.

Our results provide evidence of a persistent yet dynamic relationship between *Osedax* and specific Campylobacterales epibionts that possess unique genomic features. However, the role of the biofilm on the physiology of *Osedax* remains unknown.

## MATERIALS AND METHODS

### Specimen collection

*Osedax* specimens were collected from a whalefall at 1,018 m depth in the Monterey Canyon off the coast of California (from 2005 to 2019), using the remotely operated vehicles (ROVs) Tiburon or Doc Ricketts (on the R/V Western Flyer), and from a whalefall at 3,239 m depth on the Davidson seamount (from 2019 to 2020), using the ROV Hercules (on the R/V Nautilus; Table 1).

The whalefall at 1,018 m in the Monterey Canyon (36.772°N/122.083°W) was implanted by the Monterey Bay Aquarium Research Institute in October 2004 (ref. 4). The whalefall on the Davidson seamount (35.582°N/122.629°W) was discovered serendipitously in October 2019. Several additional specimens of *O. frankpressi*, used for metagenomic analysis and microscopy, were collected from a natural whalefall in Monterey Canyon at 2,891 m (36.613°N/122.434°W). At the 1,018 m site, *Osedax* worms were collected between 8 and 172 mo after the carcass was first deposited on the seafloor (Table 1). Whalefall stages were categorized as being in early, mid, or late stages by the progression of bone degradation; “early” designated as having significant whale tissue and bone biomass present; “mid” designated as having little whale tissue present, and “late” stages designated by extreme reduction in bone biomass (Fig. 1). Pursuant to the Marine Mammal Protection Act (50 CFR 216.22 and 216.37), authorization was received for the Monterey Bay Aquarium Research Institute and the Monterey Bay National Marine Sanctuary (MBNMS-2020–006) to collect whalefall specimens for scientific purposes during exploratory dives via remotely operated vehicles in the Monterey Bay National Marine Sanctuary. Additionally, a general CDFW collecting permit SC-10578 (to S. Goffredi) was acquired for the collection of *Osedax* specifically. All *Osedax* species used in this study have been previously described, with the exception of one undescribed species from the Davidson Seamount from dive H1796 (Table 1).

### Microscopy

Specimens for FISH microscopy were initially preserved in 4% sucrose-buffered paraformaldehyde (PFA) and kept at 4°C. These PFA-preserved specimens were rinsed with 2 × PBS, transferred to 70% ethanol, and stored at –20°C. Tissues were dissected and embedded in Steedman’s wax [one part cetyl alcohol: nine parts polyethylene glycol (400) distearate, mixed at 60°C]. An ethanol: wax gradient of 3:1, 2:1, and 1:1, and eventually 100% wax, was used to embed the samples (1 h each treatment). Embedded samples were sectioned at 2–5 μm thickness using a Leica RM2125 microtome and placed on Superfrost Plus slides. Sections were dewaxed in 100% ethanol rinses. The hybridization buffer included 35% formamide, and fluorescent probes at final concentrations of 5 µg/mL, while the wash solution contained 450 mM NaCl (10). We used the epsilonproteobacteria-specific probe EPS549; ref. 55) labeled with FITC or Cy3. A universal bacterial probe (Eub338-I; ref. 56), labeled with Cy3, Cy5, or Alexa488, was also used. Probes were hybridized at 46°C for 4–8 h, followed by a 15 min wash at 48°C. Sections were counterstained with 4′6′-diamidino-2-phenylindole (DAPI, 5 mg/mL) for 5 min, rinsed and mounted in Citifluor, and examined by epifluorescence microscopy using a Nikon E80i epifluorescence microscope with a Nikon DS-Qi1Mc high-sensitivity monochrome digital camera.

For examination by transmission electron microscopy, samples (approximately 1 mm^3^) were fixed in 3% glutaraldehyde buffered with 0.1 M phosphate and 0.3 M sucrose (pH 7.8). Following a wash in 0.1 M sodium cacodylate containing 24% sucrose, samples were postfixed with 1% OsO4 in 0.1 M sodium cacodylate for 1 h, stained *en bloc* in 3% uranyl acetate in 0.1 M sodium acetate buffer for 1 h, dehydrated through an ethanol series, then infiltrated and embedded in Spurr’s resin (Ted Pella, Redding, CA, USA). Thin (70 nm) sections were stained with methylene blue and lead citrate, respectively, and then examined and photographed using a Zeiss Labrolux 12 light microscope and Zeiss EM109 TEM.

### Molecular analysis

Specimens for molecular analysis were either frozen at −80°C or preserved immediately upon collection in ~90% ethanol. Total genomic DNA was extracted using the Qiagen DNeasy kit (Qiagen, Valencia, CA, USA) according to the manufacturer’s instructions. For some specimens, DNA was extracted from *Osedax* trunk tissues only, while for others, as in small species like *O. talkovici*, whole specimens were used. Select mucous tubes were also extracted, separate from animal tissue.

*Osedax* identity was confirmed by sequencing the mitochondrial cytochrome c oxidase subunit I gene (COI). This gene was amplified using the previously published primers LCO1490/HCO2198, according to ref 57. Amplification products were sequenced directly using Sanger sequencing, via Laragen Inc. (Culver City, CA, USA), and compared to published sequences in GenBank and in ref. 6.

To characterize the *Osedax*-associated bacterial diversity, we performed 16S rRNA gene amplicon sequencing. The V4-V5 region of the 16S rRNA gene was amplified using bacterial primers with Illumina (San Diego, CA, USA) adapters on the 5′ ends of 515F/806R (ref. 26), with Q5 Hot Start High-Fidelity 2 x Master Mix (New England Biolabs, Ipswich, MA, USA) and annealing conditions of 54°C for 25 cycles. Each product (2.5 µL) was barcoded with Illumina NexteraXT index 2 Primers that include unique 8 bp barcodes (64°C annealing temperature and 11 cycles). Secondary amplification products were purified via vacuum manifold (Millipore-Sigma MultiScreen plates (St. Louis, MO, USA) and quantified using QuantIT PicoGreen dsDNA (Thermo-Fisher Scientific; Waltham, MA, USA) on a BioRad CFX96 Touch Real-Time PCR Detection System. Barcoded samples were combined in equimolar amounts (~100 ng) into a single tube and purified with the Promega Wizard SV Gel and PCR Clean-Up kit (#A9281) before submission to Laragen (Culver City, CA, USA) for 2 × 300 bp paired end analysis on the Illumina MiSeq platform with PhiX addition of 15–20%. MiSeq 16S rRNA gene sequence data were processed in Quantitative Insights Into Microbial Ecology (v1.8.0; ref. 58), using the default parameters. Sequences were clustered into *de novo* operational taxonomic units (OTUs) with 99% similarity using UCLUST open reference clustering protocol, and then, the most abundant sequence was chosen as representative for each *de novo* OTU. Taxonomic identification for each representative sequence was assigned using the Silva-138 database (59), clustered at 99% similarity. A threshold filter was used to remove any OTU that occurred below 0.01% in the combined samples dataset. Analyses are based on Bray-Curtis distances of fourth-root transformed data. Quantification and statistical analyses are described in the Results section and figure legends.

QPCR was carried out in order to compare the relative abundance of bacteria on a subset of *Osedax* trunks. In brief, a 154 bp partial bacterial 16S rRNA gene target was amplified using the primers 303F/457R (10). Reactions for all DNA extracts were conducted in duplicate and contained 10 µL iTaq Universal Sybr green mix (Bio-Rad), 8 µL RNase- and DNase-free deionized water, 1 µL DNA sample (normalized to 2 ng µL^-1^), and final primer concentrations of 200 nM. These primers were previously optimized for amplification efficiency using positive and negative controls (described in ref. 10). QPCR assays were run on a Bio-Rad CFX96 Touch Real-Time PCR detection system under the following thermal conditions: incubation for 2 min at 50°C and Taq activation for 3 min at 95°C, followed by 40 cycles of 15 s of denaturation at 95°C and 60 s of annealing/extension at 55°C. A dissociation curve from each QPCR reaction was examined to further ensure proper target sequence amplification. DNA abundance was calculated from the number of cycles necessary for fluorescence to exceed a set threshold value (CT) relative to standard controls with known DNA concentrations.

### Microbial genomes: DNA extraction, sequencing, and bioinformatic analysis

High molecular weight genomic DNA was extracted from an entire *Osedax frankpressi* adult female following the Bionano genomics IrysPrep agar-based animal tissue protocol (Catalogue # 80002). Sequencing of the gDNA was performed at UC Berkeley with a PacBio Sequel II machine to generate long reads of high contiguity and on an Illumina HiSeq6000 for short reads of high accuracy and depth of coverage. The reads were profiled taxonomically using Kraken (60) to filter out eukaryotic reads, and then all prokaryotic reads were co-assembled using MetaFlye (61) with automatic genome size selection followed by 10 polishing iterations. The assembly graphs were manually inspected using Bandage (62) and were binned using MaxBin2 (63) with a minimum contig length of 1,000 base-pairs maximum iteration of 50 and a probability threshold of 0.9. Each bacterial genome was then polished with both the short and long reads using NextPolish (64) following the recommended configuration. To resolve contamination issues due to heterogeneity of *Sulfurospirillum* strains within our sample, we assembled using the short Illumina reads using SPAdes v.3.15.4 (ref. 65) and used BlobTools v.1.1.1 (ref. 66) to select the most abundant *Sulfurospirillum* strain. *Sulfurospirillum* contigs based on Illumina data were then mapped and polished to long-reads using NextPolish (64), yielding a genome of high contiguity and completeness with low heterogeneity. Assembly metrics were generated using MetaQuast (67), genome completeness and contamination were checked using BUSCO (68) and CheckM (69), and their taxonomic IDs were identified using GTDB-Tk via wgANI (70) and taxonomic placement of the genomes alongside the thousands of references in the GTDB database.

The genomes were annotated using Prokka (71) using—kingdom Bacteria—gcode 11—compliant, and amino acid translations of the annotations were used in OrthoVenn2 (72) for gene enrichment analysis following default parameters. Hidden Markov Motifs (HMMs) involved in metabolism were identified using Lithogenie through the MagicLamp tool (73) utilizing the curated enzymatic motifs from K. Anantharaman (https://github.com/kanantharaman/metabolic-hmms). Insertion sequences were detected using ISSAGA (74) and IslandViewer4 (75), for all predicted transposable elements and the associated mobilized functional genes. Secreted proteins with eukaryotic- like domains were identified using EffectiveELD through EffectiveDB, on default settings (76). Secretion system proteins were annotated using TXSScan (77) through MacSyFinder using curated motifs to check for the presence of genes involved in protein secretion machinery, irrespective of order, to allow the detection of horizontally acquired genes and enriched copies of certain parts of the secretion machinery. Bacteriophages were investigated using Phaster (78) using the genome assembly of each epibiont as fasta input. One sample *T* tests to report on the significant differences in genomic features between the epibionts and free-living relatives were conducted using ggpubr v.0.1 (ref. 79). The absence of carbon, sulfur, and nitrogen metabolism genes in *Sulfurospirillum*-related reads was confirmed by screening the unbinned reads.

## Data Availability

The raw Illumina 16S rRNA gene barcode sequences and metadata collected in this study are available from the NCBI Sequence Read Archive (project number PRJNA813533). The 4 *Osedax*-specific Campylobacterales genome assemblies, as well as an Alphaproteobacteria, were deposited to Genbank under project number PRJNA813420.

## SUPPLEMENTAL MATERIAL

Supplemental material mbio.03140-22-s0001.pdf Supplemental text, Fig. S1 to S6, and Tables S1 to S5.

ASM does not own the copyrights to Supplemental Material that may be linked to, or accessed through, an article. The authors have granted ASM a non-exclusive, world-wide license to publish the Supplemental Material files. Please contact the corresponding author directly for reuse.

## References

[1] Rouse GW , Goffredi SK , Vrijenhoek RC . 2004. Osedax: bone-eating marine worms with dwarf males. Science 305:668–671. doi:10.1126/science.1098650 15286372

[2] Goffredi SK , Orphan VJ , Rouse GW , Jahnke L , Embaye T , Turk K , Lee R , Vrijenhoek RC . 2005. Evolutionary innovation: a bone-eating marine symbiosis. Environ Microbiol 7:1369–1378. doi:10.1111/j.1462-2920.2005.00824.x 16104860

[3] Tresguerres M , Katz S , Rouse GW . 2013. How to get into bones: proton pump and carbonic anhydrase in Osedax boneworms. Proc Biol Sci 280: 20130625. doi:10.1098/rspb.2013.0625 23760644 PMC3652447

[4] Braby CE , Rouse GW , Johnson SB , Jones WJ , Vrijenhoek RC . 2007. Bathymetric and temporal variation among Osedax boneworms and associated megafauna on whale-falls in Monterey Bay, California. Deep Sea Res I: Oceanogr Res Papers 54:1773–1791. doi:10.1016/j.dsr.2007.05.014

[5] Vrijenhoek RC , Johnson SB , Rouse GW . 2009. A remarkable diversity of bone-eating worms (Osedax; Siboglinidae; Annelida). BMC Biol 7:1–3. doi:10.1186/1741-7007-7-74 19903327 PMC2780999

[6] Rouse GW , Goffredi SK , Johnson SB , Vrijenhoek RC . 2018. An inordinate fondness for Osedax (Siboglinidae: Annelida): fourteen new species of bone worms from California. Zootaxa 4377:451–489. doi:10.11646/zootaxa.4377.4.1 29690036

[7] Salathé RM , Vrijenhoek RC . 2012. Temporal variation and lack of host specificity among bacterial endosymbionts of Osedax bone worms (Polychaeta: Siboglinidae). BMC Evol Biol 12:189. doi:10.1186/1471-2148-12-189 23006795 PMC3551747

[8] Goffredi SK , Yi H , Zhang Q , Klann JE , Struve IA , Vrijenhoek RC , Brown CT . 2014. Genomic versatility and functional variation between two dominant heterotrophic symbionts of deep-sea Osedax worms. ISME J 8:908–924. doi:10.1038/ismej.2013.201 24225886 PMC3960542

[9] Goffredi SK , Wilpiszeski R , Lee R , Orphan VJ . 2008. Temporal evolution of methane cycling and phylogenetic diversity of archaea in sediments from a deep-sea whale-fall in Monterey Canyon, California. ISME J 2:204–220. doi:10.1038/ismej.2007.103 18219285

[10] Goffredi SK , Johnson SB , Vrijenhoek RC . 2007. Genetic diversity and potential function of microbial symbionts associated with newly discovered species of Osedax polychaete worms. Appl Environ Microbiol 73:2314–2323. doi:10.1128/AEM.01986-06 17277220 PMC1855680

[11] Verna C , Ramette A , Wiklund H , Dahlgren TG , Glover AG , Gaill F , Dubilier N . 2010. High symbiont diversity in the bone‐eating worm Osedax mucofloris from shallow whale‐falls in the North Atlantic. Environ Microbiol 12:2355–2370. doi:10.1111/j.1462-2920.2010.02299.x 21966925

[12] Fujikura K , Fujiwara Y , Kawato M . 2006. A new species of Osedax (Annelida: Siboglinidae) associated with whale carcasses off Kyushu, Japan. Zoolog Sci 23:733–740. doi:10.2108/zsj.23.733 16971793

[13] Nakagawa S , Takai K , Inagaki F , Hirayama H , Nunoura T , Horikoshi K , Sako Y . 2005. Distribution, phylogenetic diversity and physiological characteristics of -proteobacteria in a deep-sea hydrothermal field. Environ Microbiol 7:1619–1632. doi:10.1111/j.1462-2920.2005.00856.x 16156735

[14] Campbell BJ , Engel AS , Porter ML , Takai K . 2006. The versatile ε-proteobacteria: key players in sulphidic habitats. Nat Rev Microbiol 4:458–468. doi:10.1038/nrmicro1414 16652138

[15] Hügler M , Gärtner A , Imhoff JF . 2010. Functional genes as markers for sulfur cycling and Co2 fixation in microbial communities of hydrothermal vents of the Logatchev field. FEMS Microbiol Ecol 73:526–537. doi:10.1111/j.1574-6941.2010.00919.x 20597983

[16] Nakagawa T , Takai K , Suzuki Y , Hirayama H , Konno U , Tsunogai U , Horikoshi K . 2006. Geomicrobiological exploration and characterization of a novel deep‐sea hydrothermal system at the TOTO caldera in the mariana volcanic arc. Environ Microbiol 8:37–49. doi:10.1111/j.1462-2920.2005.00884.x 16343320

[17] Huber JA , Cantin HV , Huse SM , Welch DBM , Sogin ML , Butterfield DA . 2010. Isolated communities of Epsilonproteobacteria in hydrothermal vent fluids of the Mariana Arc seamounts. FEMS Microbiol Ecol 73:538–549. doi:10.1111/j.1574-6941.2010.00910.x 20533947

[18] McNichol J , Stryhanyuk H , Sylva SP , Thomas F , Musat N , Seewald JS , Sievert SM . 2018. Primary productivity below the seafloor at Deep-Sea hot springs. Proc Natl Acad Sci U S A 115:6756–6761. doi:10.1073/pnas.1804351115 29891698 PMC6042141

[19] Tringe SG , von Mering C , Kobayashi A , Salamov AA , Chen K , Chang HW , Podar M , Short JM , Mathur EJ , Detter JC , Bork P , Hugenholtz P , Rubin EM . 2005. Comparative metagenomics of microbial communities. Science 308:554–557. doi:10.1126/science.1107851 15845853

[20] Goffredi SK , Orphan VJ . 2010. Bacterial community shifts in taxa and diversity in response to localized organic loading in the deep sea. Environ Microbiol 12:344–363. doi:10.1111/j.1462-2920.2009.02072.x 19799620

[21] Wahl M , Goecke F , Labes A , Dobretsov S , Weinberger F . 2012. The second skin: ecological role of epibiotic biofilms on marine organisms. Front Microbiol 3:292. doi:10.3389/fmicb.2012.00292 22936927 PMC3425911

[22] Egan S , Gardiner M . 2016. Microbial dysbiosis: rethinking disease in marine ecosystems. Front Microbiol 7:991. doi:10.3389/fmicb.2016.00991 27446031 PMC4914501

[23] Grossart HP , Tang KW . 2010. www.aquaticmicrobial.net. Commun Integr Biol 3:491–494. doi:10.4161/cib.3.6.12975 21331222 PMC3038046

[24] Bessette S , Fagervold SK , Romano C , Martin D , Bris NL , Galand PE . 2014. Diversity of bacterial communities on sunken woods in the Mediterranean sea. J Mar SCI Tech 22:7. doi:10.6119/JMST-013-0829-2

[25] Takai K , Suzuki M , Nakagawa S , Miyazaki M , Suzuki Y , Inagaki F , Horikoshi K . 2006. Sulfurimonas paralvinellae sp. nov., a novel mesophilic, hydrogen- and sulfur-oxidizing chemolithoautotroph within the epsilonproteobacteria isolated from a deep-sea hydrothermal vent polychaete nest, reclassification of Thiomicrospira denitrificans as Sulfurimonas denitrificans comb. nov. and emended description of the genus Sulfurimonas. Int J Syst Evol Microbiol 56:1725–1733. doi:10.1099/ijs.0.64255-0 16901999

[26] Caporaso JG , Lauber CL , Walters WA , Berg-Lyons D , Lozupone CA , Turnbaugh PJ , Fierer N , Knight R . 2011. Global patterns of 16S rRNA gene diversity at a depth of millions of sequences per sample. Proc Natl Acad Sci U S A 108 Suppl 1:4516–4522. doi:10.1073/pnas.1000080107 20534432 PMC3063599

[27] Reynolds D , Thomas T . 2016. Evolution and function of eukaryotic-like proteins from sponge symbionts. Mol Ecol 25:5242–5253. doi:10.1111/mec.13812 27543954

[28] Frank AC . 2019. Molecular host mimicry and manipulation in bacterial symbionts. FEMS Microbiol Lett 366:fnz038. doi:10.1093/femsle/fnz038 30877310

[29] Hinzke T , Kleiner M , Breusing C , Felbeck H , Häsler R , Sievert SM , Schlüter R , Rosenstiel P , Reusch TBH , Schweder T , Markert S . 2019. Host-microbe interactions in the chemosynthetic Riftia Pachyptila symbiosis. mBio 10:e02243-19. doi:10.1128/mBio.02243-19 31848270 PMC6918071

[30] Díez-Vives C , Moitinho-Silva L , Nielsen S , Reynolds D , Thomas T . 2017. Expression of eukaryotic‐like protein in the microbiome of sponges. Mol Ecol 26:1432–1451. doi:10.1111/mec.14003 28036141

[31] Dahlgren TG , Wiklund H , Kallstrom B , Lundalv T , Smith CR , Glover AG . 2006. A shallow-water whale-fall experiment in the North Atlantic. Cah Biol Mar 47:385–389.

[32] Wirsen CO , Sievert SM , Cavanaugh CM , Molyneaux SJ , Ahmad A , Taylor LT , DeLong EF , Taylor CD . 2002. Characterization of an autotrophic sulfide-oxidizing marine Arcobacter sp. that produces filamentous sulfur. Appl Environ Microbiol 68:316–325. doi:10.1128/AEM.68.1.316-325.2002 11772641 PMC126556

[33] Kalenitchenko D , Dupraz M , Le Bris N , Petetin C , Rose C , West NJ , Galand PE . 2016. Ecological succession leads to chemosynthesis in mats colonizing wood in sea water. ISME J 10:2246–2258. doi:10.1038/ismej.2016.12 26905628 PMC4989304

[34] Sievert SM , Hugler M , Taylor CD , Wirsen CO . 2008. Sulfur oxidation at deep-sea Hydrothermal vents, In Dahl C , CG Friedrich (ed), Microbial sulfur metabolism. Springer, Berlin. doi:10.1007/978-3-540-72682-1

[35] McNichol J , Dyksma S , Mußmann M , Seewald JS , Sylva SP , Sievert SM . 2022. Genus-specific carbon fixation activity measurements reveal distinct responses to oxygen among hydrothermal vent Campylobacteria. Appl Environ Microbiol 88:e0208321. doi:10.1128/AEM.02083-21 34788061 PMC8788762

[36] Treude T , Smith CR , Wenzhöfer F , Carney E , Bernardino AF , Hannides AK , Krüger M , Boetius A . 2009. Biogeochemistry of a deep-sea whale fall: sulfate reduction, sulfide efflux and methanogenesis. Mar Ecol Prog Ser 382:1–21. doi:10.3354/meps07972

[37] Hamann E , Gruber-Vodicka H , Kleiner M , Tegetmeyer HE , Riedel D , Littmann S , Chen J , Milucka J , Viehweger B , Becker KW , Dong X , Stairs CW , Hinrichs K-U , Brown MW , Roger AJ , Strous M . 2016. Environmental breviatea harbour mutualistic Arcobacter epibionts. Nature 534:254–258. doi:10.1038/nature18297 27279223 PMC4900452

[38] Davidson SK , Koropatnick TA , Kossmehl R , Sycuro L , McFall-Ngai MJ . 2004. NO means ‘yes’ in the squid‐Vibrio symbiosis: nitric oxide (NO) during the initial stages of a beneficial association. Cell Microbiol 6:1139–1151. doi:10.1111/j.1462-5822.2004.00429.x 15527494

[39] Goris T , Diekert G . 2016. The genus Sulfurospirillum, p 209–234. In Adrian L , FE Löffler (ed), Organohalide-Respiring bacteria. Springer-Verlag, Berlin, Heidelberg. doi:10.1007/978-3-662-49875-0

[40] Campbell BJ , Jeanthon C , Kostka JE , Luther GW , Cary SC . 2001. Growth and phylogenetic properties of novel bacteria belonging to the epsilon subdivision of the proteobacteria enriched from Alvinella pompejana and deep-sea hydrothermal vents. Appl Environ Microbiol 67:4566–4572. doi:10.1128/AEM.67.10.4566-4572.2001 11571157 PMC93204

[41] Isaac A , Francis B , Amann RI , Amin SA . 2021. Tight adherence (tad) Pilus genes indicate putative niche differentiation in phytoplankton bloom associated rhodobacterales. Front Microbiol 12:718297. doi:10.3389/fmicb.2021.718297 34447362 PMC8383342

[42] Pu M , Duriez P , Arazi M , Rowe-Magnus DA . 2018. A conserved tad pilus promotes vibrio vulnificus oyster colonization. Environ Microbiol 20:828–841. doi:10.1111/1462-2920.14025 29235706

[43] Wang S , Jiang L , Hu Q , Cui L , Zhu B , Fu X , Lai Q , Shao Z , Yang S . 2021. Characterization of Sulfurimonas hydrogeniphila sp. nov., a novel bacterium predominant in deep-sea hydrothermal vents and comparative genomic analyses of the genus Sulfurimonas. Front Microbiol 12:626705. doi:10.3389/fmicb.2021.626705 33717015 PMC7952632

[44] Meyer JL , Akerman NH , Proskurowski G , Huber JA . 2013. Microbiological characterization of post-eruption "snowblower" vents at axial seamount, Juan de Fuca Ridge. Front Microbiol 4:153. doi:10.3389/fmicb.2013.00153 23785361 PMC3683637

[45] van der Stel A-X , Wösten M . 2019. Regulation of respiratory pathways in campylobacterota: a review. Front Microbiol 10:1719. doi:10.3389/fmicb.2019.01719 31417516 PMC6682613

[46] de Campos SB , Lardi M , Gandolfi A , Eberl L , Pessi G . 2017. Mutations in two Paraburkholderia Phymatum type VI secretion systems cause reduced fitness in interbacterial competition. Front Microbiol 8:2473. doi:10.3389/fmicb.2017.02473 29312183 PMC5732942

[47] Speare L , Woo M , Bultman KM , Mandel MJ , Wollenberg MS , Septer AN . 2021. Host-like conditions are required for T6SS-mediated competition among Vibrio fischeri light organ symbionts. mSphere 6:e0128820. doi:10.1128/mSphere.01288-20 34287008 PMC8386388

[48] Ott J , Bright M , Bulgheresi S . 2004. Symbioses between marine nematodes and sulfur-oxidizing chemoautotrophic bacteria. Symbiosis 36:103–126.

[49] Thurber AR , Jones WJ , Schnabel K . 2011. Dancing for food in the deep sea: bacterial farming by a new species of yeti crab. PLoS One 6:e26243. doi:10.1371/journal.pone.0026243 22140426 PMC3227565

[50] Volland JM , Schintlmeister A , Zambalos H , Reipert S , Mozetič P , Espada-Hinojosa S , Turk V , Wagner M , Bright M . 2018. NanoSIMS and tissue autoradiography reveal symbiont carbon fixation and organic carbon transfer to giant ciliate host. ISME J 12:714–727. doi:10.1038/s41396-018-0069-1 29426952 PMC5854253

[51] Seah BKB , Antony CP , Huettel B , Zarzycki J , Schada von Borzyskowski L , Erb TJ , Kouris A , Kleiner M , Liebeke M , Dubilier N , Gruber-Vodicka HR . 2019. Sulfur-oxidizing symbionts without canonical genes for autotrophic CO_2_ fixation. mBio 10:e01112-19. doi:10.1128/mBio.01112-19 31239380 PMC6593406

[52] Paredes GF , Viehboeck T , Lee R , Palatinszky M , Mausz MA , Reipert S , Schintlmeister A , Maier A , Volland J-M , Hirschfeld C , Wagner M , Berry D , Markert S , Bulgheresi S , König L . 2021. Anaerobic sulfur oxidation underlies adaptation of a chemosynthetic symbiont to oxic-anoxic interfaces. mSystems 6:e0118620. doi:10.1128/mSystems.01186-20 34058098 PMC8269255

[53] Borchert E , García-Moyano A , Sanchez-Carrillo S , Dahlgren TG , Slaby BM , Bjerga GEK , Ferrer M , Franzenburg S , Hentschel U . 2021. Deciphering a marine bone-degrading microbiome reveals a complex community effort. mSystems 6:e01218-20. doi:10.1128/mSystems.01218-20 33563781 PMC7883544

[54] Savage DC . 1985. Effects on host animals of bacteria adhering to epithelial surfaces, In Fletcher M , DC Savage (ed), Bacterial adhesion: Mechanisms and physiological significance. Springer, Boston, MA. doi:10.1007/978-1-4615-6514-7

[55] Lin X , Wakeham SG , Putnam IF , Astor YM , Scranton MI , Chistoserdov AY , Taylor GT . 2006. Comparison of vertical distributions of prokaryotic assemblages in the anoxic Cariaco Basin and Black Sea by use of fluorescence in situ hybridization. Appl Environ Microbiol 72:2679–2690. doi:10.1128/AEM.72.4.2679-2690.2006 16597973 PMC1449015

[56] Daims H , Brühl A , Amann R , Schleifer KH , Wagner M . 1999. The domain-specific probe EUB338 is insufficient for the detection of all bacteria: development and evaluation of a more comprehensive probe set. Syst Appl Microbiol 22:434–444. doi:10.1016/S0723-2020(99)80053-8 10553296

[57] Folmer O , Black M , Hoeh W , Lutz R , Vrijenhoek R . 1994. DNA primers for amplification of mitochondrial cytochrome C oxidase subunit I from diverse metazoan invertebrates. Mol Mar Biol Biotechnol 3:294–299. 7881515

[58] Caporaso JG , Kuczynski J , Stombaugh J , Bittinger K , Bushman FD , Costello EK , Fierer N , Peña AG , Goodrich JK , Gordon JI , Huttley GA , Kelley ST , Knights D , Koenig JE , Ley RE , Lozupone CA , McDonald D , Muegge BD , Pirrung M , Reeder J , Sevinsky JR , Turnbaugh PJ , Walters WA , Widmann J , Yatsunenko T , Zaneveld J , Knight R . 2010. QIIME allows analysis of high-throughput community sequencing data. Nat Methods 7:335–336. doi:10.1038/nmeth.f.303 20383131 PMC3156573

[59] Pruesse E , Quast C , Knittel K , Fuchs BM , Ludwig W , Peplies J , Glöckner FO . 2007. SILVA: a comprehensive online resource for quality checked and aligned ribosomal RNA sequence data compatible with ARB. Nucleic Acids Res 35:7188–7196. doi:10.1093/nar/gkm864 17947321 PMC2175337

[60] Wood DE , Salzberg SL . 2014. Kraken: ultrafast metagenomic sequence classification using exact alignments. Genome Biol 15:1–2. doi:10.1186/gb-2014-15-3-r46 PMC4053813 24580807

[61] Kolmogorov M , Bickhart DM , Behsaz B , Gurevich A , Rayko M , Shin SB , Kuhn K , Yuan J , Polevikov E , Smith TPL , Pevzner PA . 2020. MetaFlye: scalable long-read metagenome assembly using repeat graphs. Nat Methods 17:1103–1110. doi:10.1038/s41592-020-00971-x 33020656 PMC10699202

[62] Wick RR , Schultz MB , Zobel J , Holt KE . 2015. Bandage: interactive visualization of de novo genome assemblies. Bioinformatics 31:3350–3352. doi:10.1093/bioinformatics/btv383 26099265 PMC4595904

[63] Wu YW , Simmons BA , Singer SW . 2016. Maxbin 2.0: an automated binning algorithm to recover genomes from multiple metagenomic datasets. Bioinformatics 32:605–607. doi:10.1093/bioinformatics/btv638 26515820

[64] Hu J , Fan J , Sun Z , Liu S . 2020. Nextpolish: a fast and efficient genome polishing tool for long-read assembly. Bioinformatics 36:2253–2255. doi:10.1093/bioinformatics/btz891 31778144

[65] Bankevich A , Nurk S , Antipov D , Gurevich AA , Dvorkin M , Kulikov AS , Lesin VM , Nikolenko SI , Pham S , Prjibelski AD , Pyshkin AV , Sirotkin AV , Vyahhi N , Tesler G , Alekseyev MA , Pevzner PA . 2012. Spades: a new genome assembly algorithm and its applications to single-cell sequencing. J Comput Biol 19:455–477. doi:10.1089/cmb.2012.0021 22506599 PMC3342519

[66] Laetsch DR , Blaxter ML . 2017. Blobtools: interrogation of genome assemblies. F1000Res 6:1287. doi:10.12688/f1000research.12232.1

[67] Mikheenko A , Saveliev V , Gurevich A . 2016. Metaquast: evaluation of metagenome assemblies. Bioinformatics 32:1088–1090. doi:10.1093/bioinformatics/btv697 26614127

[68] Simão FA , Waterhouse RM , Ioannidis P , Kriventseva EV , Zdobnov EM . 2015. BUSCO: assessing genome assembly and annotation completeness with single-copy orthologs. Bioinformatics 31:3210–3212. doi:10.1093/bioinformatics/btv351 26059717

[69] Parks DH , Imelfort M , Skennerton CT , Hugenholtz P , Tyson GW . 2015. Checkm: assessing the quality of microbial genomes recovered from isolates, single cells, and metagenomes. Genome Res 25:1043–1055. doi:10.1101/gr.186072.114 25977477 PMC4484387

[70] Chaumeil P-A , Mussig AJ , Hugenholtz P , Parks DH , Hancock J . 2019. GTDB-TK: a toolkit to classify genomes with the genome taxonomy database. Bioinformatics 36:1925–1927. doi:10.1093/bioinformatics/btz848 31730192 PMC7703759

[71] Seemann T . 2014. Prokka: rapid prokaryotic genome annotation. Bioinformatics 30:2068–2069. doi:10.1093/bioinformatics/btu153 24642063

[72] Xu L , Dong Z , Fang L , Luo Y , Wei Z , Guo H , Zhang G , Gu YQ , Coleman-Derr D , Xia Q , Wang Y . 2019. OrthoVenn2: a web server for whole-genome comparison and annotation of orthologous clusters across multiple species. Nucleic Acids Res 47:W52–W58. doi:10.1093/nar/gkz333 31053848 PMC6602458

[73] Garber AI , Ramirez GA , Merino N , Pavia MJ , McAllister SM . 2020. Magiclamp: toolkit for annotation of 'omics datasets using curated HMM sets. 2021: Magiclamp, Github repository. Available from: https://github.com/Arkadiy-Garber/MagicLamp

[74] Varani AM , Siguier P , Gourbeyre E , Charneau V , Chandler M . 2011. Issaga is an ensemble of web-based methods for high throughput identification and semi-automatic annotation of insertion sequences in prokaryotic genomes. Genome Biol 12:R30. doi:10.1186/gb-2011-12-3-r30 21443786 PMC3129680

[75] Bertelli C , Laird MR , Williams KP , Simon Fraser University Research Computing Group, Lau BY , Hoad G , Winsor GL , Brinkman FSL . 2017. Islandviewer 4: expanded prediction of genomic Islands for larger-scale datasets. Nucleic Acids Res 45:W30–W35. doi:10.1093/nar/gkx343 28472413 PMC5570257

[76] Eichinger V , Nussbaumer T , Platzer A , Jehl M-A , Arnold R , Rattei T . 2016. EffectiveDB--updates and novel features for a better annotation of bacterial secreted proteins and type III, IV, VI secretion systems. Nucleic Acids Res 44:D669–D674. doi:10.1093/nar/gkv1269 26590402 PMC4702896

[77] Abby SS , Cury J , Guglielmini J , Néron B , Touchon M , Rocha EPC . 2016. Identification of protein secretion systems in bacterial genomes. Sci Rep 6:23080. doi:10.1038/srep23080 26979785 PMC4793230

[78] Arndt D , Grant JR , Marcu A , Sajed T , Pon A , Liang Y , Wishart DS . 2016. PHASTER: a better, faster version of the PHAST phage search tool. Nucleic Acids Res 44:W16–W21. doi:10.1093/nar/gkw387 27141966 PMC4987931

[79] Kassambara A , Kassambara MA . 2020. Package ‘ggpubr'. R package version 0.1. 2020;6.

